# Under nutrition as a predictor of poor academic performance; the case of Nekemte primary schools students, Western Ethiopia

**DOI:** 10.1186/s13104-019-4771-5

**Published:** 2019-11-06

**Authors:** Dejene Seyoum, Reta Tsegaye, Amanuel Tesfaye

**Affiliations:** 1 Institute of Health Sciences, Wollega University, Nekemte, Ethiopia; 2 Rift Valley University, Nekemte, Ethiopia

**Keywords:** Under nutrition, Academic performance, Nekemte, Western Ethiopia

## Abstract

**Objective:**

Though gradual improvements are exist; Ethiopia’s learning outcomes are still low in primary schools. Academic achievement of school age children can be affected by several factors such as nutritional status, socio-economic and demographic factors. The aim of this study was to assess predictors of poor academic performance in Nekemte Primary school students, Western Ethiopia.

**Results:**

A total of 362 schoolchildren and their parents were involved in the study. The study involved interviewing the participants and their parents, anthropometric measurement of participants and their document review. The prevalence of stunting and underweight was 30.2% and 45.9% respectively. Of the total study participants, 32.2% of them were poor in academic achievement. Variables like Being underweight (Adjusted odds ratio (AOR): 0.57; 95% Confidence interval (CI) 0.23–0.82), Skipping breakfast (AOR: 2.1; 95% CI 1.42–5.76), stunting (AOR: 0.66; 95% CI 0.12–0.93), being male (AOR: 2.8; 95% CI 1.79–4.52), participants whom mothers didn’t attend formal education (AOR: 0.62; 95% CI 0.37–0.92) were significantly associated with academic performance. Thus, Modifiable factors like under nutrition should be a great concern to improve the overall achievement of children in schooling.

## Introduction

Education is a key factor for development and can also be a burden for a country if there is high failure rate, since it consumes much resource from a country. Besides, all parents want their children to be smart and intellectual; therefore school failure is stress full for both parents and students. Children are considered the greatest national resource of any country who build the future of the nation, and schooling is an instrument of individual and social change, increasing the probabilities of general well-being [1, 2].

Though gradual improvements are exist, Ethiopia’s learning outcomes are still low in primary schools. For instance, over a half of 4th grade and 8th grade students tested in 2015 scored below proficiency level defined as “Below Basic” in all subjects, except English for grade 8. This means that the student has minimal understanding of the subject and lacks the skills to solve simple problems appropriate for that grade level [3]. Academic achievement of school age children can be affected by several factors such as nutritional status, demographics, and socio-economic factors [4].

Several studies have shown that malnutrition negatively affects the academic performance of students [5–10]. The consequences of malnutrition should be a significant concern for policymakers in Ethiopia, where about 5.8 million children under 5 years (38%) are suffering from chronic malnutrition (stunting or low height-for-age), according to the most recent Demographic and Health Survey (DHS) [11]. A cross sectional survey conducted in southern Ethiopia revealed that the prevalence of stunting was 16.9% and its correlation with school underperformance was significantly high [12]. Similarly studies conducted in Goba town and primary schools of Hawa Galan district shown that nutritional status like stunting, underweight and wealth index were found to be correlated with academic performance of students [4, 13]. On the other hand, there are some studies which revealed that there was no significant association between academic performance and nutritional status [1, 14].

Despite the existence of studies conducted on prevalence of under nutrition and its correlation with academic performance in Ethiopia, it is still limited to certain settings and varying associations between under nutrition and academic performance. In the current study area, there is no previous published study on academic performance of primary schools and its association with nutritional status. Therefore, this study was aimed to assess factors associated with poor academic performance in Nekemte Primary school students, Western Ethiopia.

## Main text

### Methods

#### Study design, period and setting

An institution based cross-sectional study was conducted from April to May, 2016 in Nekemte full cycle primary schools. Nekemte is an administrative capital of the East Wollega Zone located 328 km to the West of Addis Ababa, the capital city of Ethiopia. It has a latitude and longitude of 9°5′ N 36°33′ E/9.083° N 36.550° E and elevation of 2088 m. According to the 2007 national census, it has a population of 75,219 (38,385 males, 36,834 females) [15].

#### Population and sampling

A stratified sampling technique was employed to select the study units from the schools. First the selected full cycle primary school students were stratified based on their grades. Then proportional allocation was made for each grade. Study units were selected by using simple random sampling. Students list were obtained from the schools and used as a sampling frame. The sample size was determined by using single population proportion formula, ***n ***=Zα/2^*2*^ *(pq)/d*^*2*^ taking prevalence of stunting (38%) from national nutrition strategy 2010 to calculate for P giving any particular outcome to be within 5% marginal error and 95% CI of certainty (α = 05). Based on this Assumption the actual sample size for study was 362.

##### Data collection

Data were collected by interview administered questionnaires for both parents and children jointly while accompanying their children to school. Socio demographic variables of children and parents were collected from the parents themselves. The data were collected by four nursing diploma holders and data quality was insured by training them and strict supervision during data collection. Pre testing on 10% of the sample size was conducted prior to the main data collection at another school different from the sampled schools. Document review was also done by principal investigator to identify students’ average grade report of academic year 2015–2016. Academic performance was assessed by adding all subjects given in two consecutive semesters of the academic year and the average score of each student was computed. The data collection procedure also included anthropometric measurement of school children measured in separate class at their school. Weight of students was measured using beam balance with light closing, and height of students was measured to the nearest 1 cm on standing position without shoes and frequent calibrating of the scale was done. All measurements were taken twice and the mean value was used for data analysis.

#### Measurement of academic achievements

All subjects that the students were given in the academic year 2015/16 were considered to determine the academic performance of the students. Average score was calculated by taking the result of two consecutive semesters of the above-mentioned year. To examine the effect of nutritional status on educational performance, their annual average were divided into two categories; poor score and good score based on the mean value of their annual average.

#### Data management and statistical analysis

Sex, age, height and weight transferred to WHO Anthro plus software to convert nutritional data into Z-scores of the indices. To determine nutritional status of the children, the age and sex-specific growth charts were used. Height for age Z-scores (HAZ) below − 2 SD of the reference population indicates stunting. Weight for height Z-scores (WHZ) below − 2 SD of the reference population indicates wasting. Weight for age Z-scores (WAZ) below − 2 SD of the reference population is underweight [16]. Data were checked for completeness, coded and entered into SPSS version 21.0 software for analysis. Frequency tables, proportions and summarizing using texts were used to present study results. Analysis was done by using univariate and multivariable logistic regression model. Variables with a P-value less or equal to 0.2 in the univariate analysis were included in the multivariable logistic regression model. Finally, significant associations between good academic performance and independent variables with 95% confidence interval were determined at P-value less than 0.05.

#### Study variables

The dependent variable in this study was academic performance (average grade score of students) as poor and good academic performance Those whose annual grade average below the mean were regarded as poor and those whose annual grade average above the mean were termed as good academic performers. The independent variables for this study include age, sex, educational level of parents, occupation of parents, participants’ nutritional status variables like underweight, stunting and wasting.

### Results

#### Socio-demographic characteristics of the respondents and parents

A total of 362 students were enrolled into this study. One hundred sixty-nine (46.6%) of them were males and 193 (53.2%) females. The mean (SD) ages of respondents were 12.9 (± 2.2) years. Majority (80.2%) were in the age range of 10–14 and Oromo in ethnicity (84.8%). Three hundred twenty-nine, 329 (90.6%) of the participants live in urban kebeles of Nekemte town. The majority of their fathers (81%) were at least able to read and write while only 17.4% of their mothers were able to read and write. More than three-fifth of parents were either government or private employee and 59% of families of the study participants live in their own house (Table 1).

**Table 1:** Socio-demographic variables of respondents and their parents in Nekemte primary schools, Western Ethiopia, 2016 (N = 362)

Variables	Frequency	Percent
Grade		
Five	102	28.1
Six	88	24.2
Seven	91	25.1
Eight	81	22.3
Sex		
Male	169	46.6
Female	193	53.2
Age group		
10–14	291	80.2
15–19	71	19.6
Residence		
Urban	329	90.6
Rural	33	9.1
Religion		
Orthodox	150	41.3
Muslim	48	13.3
Protestant	117	32.2
Other	47	12.9
Ethnicity		
Oromo	308	85.01
Amhara	37	10.2
Others^a^	17	4.7
Number of people living in the house		
2 to 4	108	29.8
5 to 12	254	70.2
Mothers educational status		
Unable to read and write	79	21.8
Read and write	63	17.4
Completed primary	67	18.5
Completed secondary	24	6.6
College and above	129	35.5
Fathers educational status		
Unable to read and write	69	19
Read and write	88	24.2
Completed primary	14	3.9
Completed secondary	127	35
College and above	64	17.6
Mothers occupation		
House wife	121	33.3
Farmer	17	4.7
Merchant	78	2.1
Government employee	79	21.8
Private employee/NGO	36	9.9
Daily laborer	31	8.5
Fathers occupation		
Farmer	10	2.8
Merchant	108	29.8
Government employee	73	20.1
Private employee/NGO	150	41.3
Daily laborer	21	5.8
House ownership		
Private	214	59
Rent	148	41
Number of rooms in the house		
1 to 3	161	44.1
4 and above	201	55.5
Latrine		
Yes	350	96.4
No	12	3.3

^a^Guraghe, Tigre

#### Nutritional status

##### Anthropometric assessment

Based on WHO reference standards, the prevalence of underweight was 45.9%. Students’ height for age was also calculated. Accordingly; those students with height for age below reference standard was 30.2%.

#### Academic performance

The mean score for the study participants was 64.37 with standard deviation of 9.63. Of the total study participants, 117 (32.2%) of them scored below the mean value and the 67.8% of them scored above the mean value. Being underweight was one of the anthropometric measurement affected the students’ academic performance (AOR: 0.57; 95% CI 0.23–0.82). Skipping breakfast (AOR: 2.1; 95% CI 1.42–5.76) was also associated negatively with the academic outcome of the student. Children who were stunted (AOR: 0.66; 95% CI 0.12–0.93) and underweight (AOR: 0.57; 95% CI 0.23–0.82) were less likely to have good academic performance. Participants’ and parents’ socio-demographic variables were also predictors of poor academic performance of these school children. Male participants were 2.8 times more likely to be good academic performers (AOR: 2.8; 95% CI 1.79–4.52). Those participants whom mothers didn’t attend formal education (AOR: 0.62; 95% CI 0.37–0.92) were less likely to be a good academic performer compared to their counterparts (Table 2). Residence, workload, occupation of family and child wasting were not associated with school performance in this study.

**Table 2:** Factors associated with academic performance at primary schools of Nekemte Town, Western Ethiopia, 2016 (N = 362)

Variables	Academic performance		Univariate OR (95% CI)	Multivariable OR (95% CI)	P-value
	Good	Poor			
Sex					
Male	113 (31.2)	56 (15.5)	2.85 (1.82, 4.62)	2.8 (1.79, 4.52)*	0.0001
Female	80 (22.1)	113 (31.2)	1.00	1.00	
Residence					
Urban	265 (73.2)	64 (17.7)	3 (1.15, 8.11)	1.84 (0.83–2.65)	0.443
Rural	19 (5.2)	14 (3.9)	1.00	1.00	
Extra workload					
Yes	46	65	0.64 (0.13–0.89)	0.88 (0.75–1.06)	0.155
No	127	114	1.00	1.00	
Mother education					
No formal educ.	69 (19.06)	74 (20.4)	0.9 (0.76, 1.01)	0.62 (0.37, 0.92)*	0.036
Attended formal ed.	112 (30.9)	108 (29.8)	1.00	1.00	
Mother occupation					
House wife	75 (20.7)	46 (12.7)	1.32 (1.18, 1.51)	1.28 (0.92–2.13)	0.365
Paid work	133 (36.7)	108 (29.8)	1.00	1.00	
Skip breakfast					
No	173 (47.5)	126 (34.8)	1.9 (1.34–3.20)	*2.1 (1.42*–5.76)*	0.026
Yes	27 (7.5)	38 (10.5)	1.00	1.00	
Child stunted					
Yes	51 (14.1)	58 (16.0)	0.61 (0.23–0.91)	*0.66 (0.12*–0.93)*	0.042
No	149 (41.2)	104 (28.7)	1.00	1.00	
Underweight					
No	124 (34.2)	72 (19.9)	1.00	1.00	
Yes	78 (21.5)	88 (24.3)	0.52 (0.27–0.78)	*0.57 (0.23*–0.82)*	0.033

* Statistically significant association

### Discussion

The goal of this study was to assess the nutritional status and associated factors affecting academic performance of children in primary schools of Nekemte, Ethiopia. The prevalence of stunting and underweight in the study area was 30.2% and 45.7% respectively. This study is similar with study conducted in Nuwara Eliya Educational Zone, India and Northern Ethiopia [1, 17] but lower than study conducted by Dawud et al. and the national estimate of 38% [11, 18]. This difference may attribute to difference in geographical setting and study period and socioeconomic level.

In the present study, stunted children had lower academic score than those non-stunted. Under weight is also another predictor of poor academic performance. Similar study conducted in Canadian school aged children, India, and Bale Goba revealed that stunted children were poor academic performers [1, 4, 5]. In this study, underweight children were 43% less in academic performance than those who were normal weight. However study conducted in Northern Ethiopia shown that there was no significant difference in academic performance based on weight for age of participants [14]. This may be attributed to non-storage of nutrients in different forms which could affect brain glucose utilization which in turn results in poor academic achievement.

Skipping breakfast was associated with poor academic performance in the present study. Children who didn’t skip their breakfast were 2 times more likely to have good academic score. The longitudinal study conducted among Australian children also revealed that over all academic achievement were higher in breakfast non-skippers than breakfast skippers [19]. A cross sectional study conducted in southern Ethiopia also shown that habit of skipping breakfast affected the cognitive performance of early adolescents [20]. This could be due to deficiency in maintaining adequate levels of glucose throughout the day which contributes to optimizing cognition.

The odd of good academic performance was observed among males than females in the current study (AOR: 2.8; 95% CI 1.79–4.52). Similarly, study conducted in primary schools of rural Malaysia revealed that boys have higher cognitive score than girls but lower in academic tests [8]. On the other hand, a study conducted on predictors of academic performance in Southwest Ethiopia showed that girls had good academic performance than boys [13]. The dissimilarity between these studies might be due to the difference in demography, time of study, and residential area (urban or rural) of the respondents. Participants’ mothers’ educational status was another factor affecting the academic performance of children. Those children who had no educated mother had poor academic performance than their counterparts (AOR: 0.62; 95% CI 0.37–0.92). Plenty of studies supports that educational status of parents independently affects the school performance of their children. Studies conducted by Firehiwot et al., Farooq et al., Eshetu et al. and Saimul et al. [13, 21–23] shown that having educated parents have positive association to good academic score of their school children.

## Limitations

The cross sectional design cannot establish causal relationship between variables. The study only assessed whether breakfast was eaten or not and didn’t include the quality of the breakfast. Further research is needed to determine whether certain breakfast types are more helpful for cognitive performance. Despite these, we used adequate sample size and mean score of students’ average as the cut-off value to say good or poor which could help the findings generalizable to primary school students.

## Data Availability

All data sets used are available within the manuscript

## Abbreviations

BMI: body mass index
EDHS: Ethiopian Demographic and Health Survey
HAZ: height for age Z-score
WAZ: weight for age Z-score
WHO: World Health Organization
